# Improvement in scalp hair growth in androgen-deficient women treated with testosterone: a questionnaire study

**DOI:** 10.1111/j.1365-2133.2011.10655.x

**Published:** 2012-02

**Authors:** RL Glaser, C Dimitrakakis, AG Messenger

**Affiliations:** Millennium Wellness Center, 228 E. Spring Valley Road, Dayton, OH 45458 and Department of Surgery, Wright State University Boonshoft School of Medicine3460 Colonel Glenn Highway, Dayton, OH 45435, U.S.A.; * 1st Department of Ob/Gyn, Athens University Medical School, 80 Vas. Sophias Street, 11528, Athens, Greece and National Institutes of HealthNICHD, Bldg 10, 10 Center Drive, Bethesda, MD 20892-1103, U.S.A.; † Department of Dermatology, Royal Hallamshire HospitalSheffield S10 2JF, U.K.

## Abstract

**Background:**

Androgens are thought to have an adverse effect on female scalp hair growth. However, our clinical experience of androgen replacement therapy in women with androgen deficiency, in which hair loss was seldom reported, led us to question this concept.

**Objectives:**

To evaluate the effect of subcutaneous testosterone therapy on scalp hair growth in female patients.

**Methods:**

A total of 285 women, treated for a minimum of 1 year with subcutaneous testosterone implants for symptoms of androgen deficiency, were asked to complete a survey that included questions on scalp and facial hair. Age, body mass index (BMI) and serum testosterone levels were examined.

**Results:**

Out of the 285 patients, 76 (27%) reported hair thinning prior to treatment; 48 of these patients (63%) reported hair regrowth on testosterone therapy (responders). Nonresponders (i.e. no reported hair regrowth on therapy) had significantly higher BMIs than responders (*P* = 0·05). Baseline serum testosterone levels were significantly lower in women reporting hair loss prior to therapy than in those who did not (*P* = 0·0001). There was no significant difference in serum testosterone levels, measured 4 weeks after testosterone implantation, between responders and nonresponders. No patient in this cohort reported scalp hair loss on testosterone therapy. A total of 262 women (92%) reported some increase in facial hair growth.

**Conclusions:**

Subcutaneous testosterone therapy was found to have a beneficial effect on scalp hair growth in female patients treated for symptoms of androgen deficiency. We propose this is due to an anabolic effect of testosterone on hair growth. The fact that no subject complained of hair loss as a result of treatment casts doubt on the presumed role of testosterone in driving female scalp hair loss. These results need to be confirmed by formal measurements of hair growth.

Thinning of hair is a common complaint in women. Its frequency in the female population increases with age. Two studies in caucasian women in the U.K. and U.S.A. reported prevalence rates of reduced hair density of 3–6% in women aged under 30 years, increasing to 29–42% in women aged ≥ 70 years.^1,^^2^ Although diverse pathologies can cause hair thinning, the most common diagnostic category is female pattern hair loss (FPHL). This typically presents as a diffuse reduction in hair density affecting the mid and frontal regions of the scalp with preservation of the frontal hairline. Although the distribution of hair loss in women is usually different from that seen in male balding (male androgenetic alopecia), the histopathological and dynamic changes in hair growth are similar, and FPHL has long been regarded as the female counterpart of male balding, giving rise to the term ‘female androgenetic alopecia’.^3^ Male balding is undoubtedly an androgen-dependent genetically determined trait.^4^ Observations in men with a genetic deficiency of 5α-reductase type 2 suggest that the active androgen in male balding is dihydrotestosterone (DHT) rather than testosterone.^5^ These men have ambiguous genitalia with normal or high levels of circulating testosterone but low DHT. They do not go bald and have sparse or absent beard growth. The importance of DHT in male balding has been confirmed by the positive response to treatment in clinical trials of 5α-reductase inhibitors.^6,^^7^

It is widely believed that androgens also have an adverse effect on scalp hair growth in women, and the idea that FPHL is an androgen-dependent trait remains prevalent. However, in our clinical experience (R.G.), treating more than 1100 female patients with over 10 000 subcutaneous testosterone pellet insertions, hair thinning has rarely been reported. In this institutional review board (IRB)-approved study, pre- and postmenopausal patients were treated with testosterone implants for symptoms of ‘relative androgen deficiency’ including: a diminished sense of well-being, dysphoric mood (sadness, depression, anxiety, irritability), fatigue, decreased libido, hot flushes, bone loss, decreased muscle strength, changes in cognition and memory, and insomnia. As part of a follow-up questionnaire aimed at assessing clinical response to therapy, patients were asked about their experience of hair loss before and after testosterone treatment.

## Subjects and methods

All patients in this study group are part of an ongoing 10-year, prospective IRB-approved trial on the effect of subcutaneous testosterone implants on the incidence of breast cancer.^8^ Pre- and postmenopausal patients were either self-referred or referred by their physician to this private, clinical practice (R.G.) for subcutaneous testosterone therapy for symptoms of relative androgen deficiency including: hot flushes, insomnia, depressive mood, irritability, anxiety, premenstrual syndrome, fatigue, memory loss, menstrual or migraine headaches, vaginal dryness, sexual problems, urinary symptoms, pain and bone loss.

Female patients treated with testosterone implant therapy for at least 1 year, seen at the clinic between February and April 2010, were accrued to this ‘follow-up’ questionnaire study. All 285 eligible patients completed the clinical survey and were offered serum testing. Written informed consent was obtained from all patients.

Patients included in this study had received treatment with subcutaneous testosterone implant therapy for 28·1 ± 10·4 months (range 12–56). Testosterone implants had been inserted, on average, every 13·8 ± 3·8 weeks (i.e. when symptoms returned). The mean testosterone implant dose in this cohort of patients was 133·3 ± 26·8 mg.

### Questionnaire

The questions on the survey regarding hair status were designed to evaluate the patient’s perception of hair ‘thinning’ (hair loss) and her clinical response to testosterone implant therapy. Two questions on the survey addressed scalp hair (Table 1). The surveys were self-completed by the patient at the time of their appointment. The medical staff was available to assist patients.

**Table 1:** Questions in survey relating to scalp hair and effects of testosterone therapy

Question	Available response
Did you have any thinning of scalp hair prior to therapy?	Yes (Y) or no (N)
If hair thinning was reported (Y), the patient was then asked to respond to the subsequent question:	
Did testosterone therapy help hair regrowth on your scalp?	Yes (Y), i.e. regrowth of hair on scalp or no (N), i.e. no regrowth of hair on scalp
An additional question addressed potential side-effects from testosterone implant therapy:	
Did you notice any negative or adverse side-effects from testosterone pellet therapy? If (Y), the patient was asked to list the side-effect	Yes (Y) or no (N)
Patients were also questioned about the effect of testosterone therapy on facial hair:	
Did facial hair increase?	No increase, minimal, moderate, severe

### Serum testosterone

Baseline serum testosterone levels were measured in 228 of the 285 patients prior to beginning testosterone therapy. Baseline testosterone levels were not available in 19 patients, and 38 patients had salivary hormone testing done. In addition, patients were encouraged, but not required, to have serum testosterone levels drawn 4 weeks after their testosterone pellets were inserted. Total testosterone levels were measured using liquid chromatography tandem mass spectrometry. The intra-assay coefficient of variation was 9%.

### Statistical analysis

The statistical program R (R Development Core Team, 2009; http://www.r-project.org/) was used for all data analysis. Age was calculated using the patient’s birth-date and the survey completion (pellet implantation) date. The Wilcoxon test was used to compare median age of patients who reported ‘hair thinning’ prior to therapy and those who did not.

Body mass index (BMI) was calculated using patients’ recorded height and weight at baseline, i.e. the initial visit, prior to testosterone therapy, and again at follow-up, i.e. at the date of the survey, on testosterone therapy.

Because hair loss is associated with insulin resistance and obesity, the Wilcoxon test with the one-sided alternative was used to determine if the distribution of BMI (baseline) was greater in the group who reported ‘hair thinning’ prior to testosterone therapy and those who did not. It was also used to compare mean BMI (follow-up) in patients who reported ‘hair regrowth’ on testosterone implant therapy and those who did not.

To investigate whether baseline testosterone levels were related to reported hair thinning, the Wilcoxon rank-sum test was employed. The Kruskal–Wallis rank sum test was used to determine if follow-up testosterone levels on therapy varied significantly among three patient cohorts: patients reporting ‘hair regrowth’ vs. ‘no hair regrowth’ vs. ‘no prior hair loss’. Where relevant, results are given as mean ± SD.

## Results

**Fig 1:**
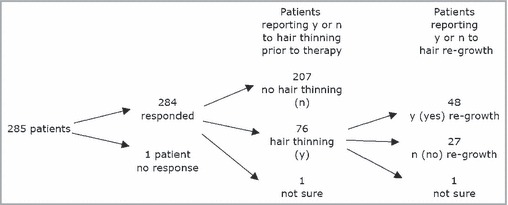
Responses to questionnaire by study participants (*n* = 285) treated with subcutaneous testosterone implants.

Out of 285 patients, 284 completed the survey (including the questions addressing scalp hair); 283 responded yes (Y) or no (N) to hair thinning prior to testosterone therapy. Only one patient was ‘not sure’ if she had hair thinning prior to therapy. Seventy-six of the 284 responders (26·8%) reported ‘hair thinning’ prior to testosterone implant therapy; 48 of these 76 patients (63·2%) reported ‘hair regrowth’ on testosterone therapy (Fig. 1). None of the subjects reported loss of hair during treatment.

Patients who reported ‘hair thinning’ prior to therapy were older (56·6 ± 7·82 years) than patients who reported ‘no hair thinning’ prior to therapy (54·1 ± 8·01 years; *W* = 6657·5, *P* = 0·05).

There was no difference in baseline BMI between women who reported hair thinning prior to testosterone therapy (25·45 ± 4·45 kg m^−2^) and those who did not (25·15 ± 4·51 kg m^−2^) (*W* = 7325, *P* = 0·20). However, patients who reported ‘no hair regrowth’ on testosterone therapy had a significantly higher BMI (26·01 ± 4·04 kg m^−2^) compared with patients who reported ‘hair regrowth’ on testosterone therapy (24·61 ± 4·24 kg m^−2^) (*W* = 814·5, *P* = 0·05). Of patients who reported ‘no hair regrowth’, 59·3% had a BMI > 25 kg m^−2^ compared with 38·3% of patients who reported ‘hair regrowth’. In addition, five of the 27 patients who reported ‘no regrowth’ on therapy had confounding conditions: Hashimoto thyroiditis (*n* = 2), thyroid-stimulating hormone (TSH) < 0·02 MIU mL^−1^ (*n* = 1), documented iron deficiency (*n* = 2). In contrast, only one of 48 patients who reported ‘hair regrowth’ on testosterone therapy had a TSH < 0·05 MIU mL^−1^ and there were no documented cases of thyroiditis or iron deficiency.

A total of 92·1% of the patients reported an increase in facial hair growth on testosterone implant therapy with the majority rating it as ‘minimal or moderate’ (85·7%) vs. ‘severe’ (6·4%). Although some patients requested a lower dose of testosterone, no one discontinued therapy for this reason.

### Serum testosterone

Baseline serum testosterone levels were available for 228 of 285 patients. Follow-up testosterone levels, measured 4 weeks after testosterone pellet implantation, were available on 154 patients.

Baseline serum testosterone levels were significantly lower in women who reported ‘hair thinning’ (14·18 ± 10·51 ng dL^−1^) compared with women who reported ‘no hair thinning’ (21·92 ± 15·69 ng dL^−1^) prior to testosterone implant therapy (*W* = 6966, *P* = 0·0001).

**Fig 2:**
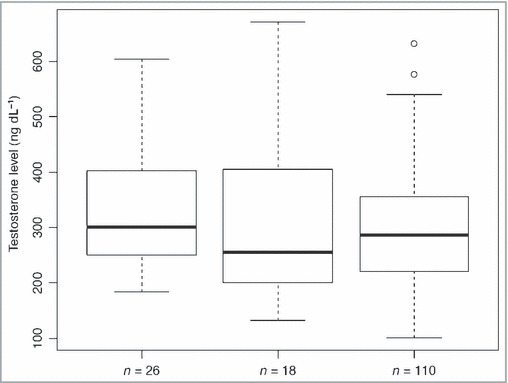
Week-4 serum testosterone levels measured in 26 of 48 patients reporting ‘hair regrowth’ on testosterone implant therapy, 18 of 27 patients reporting ‘no hair regrowth’ on testosterone implant therapy and 110 of 195 patients reporting ‘no hair thinning’ prior to testosterone therapy.

There was no significant difference in serum testosterone levels, measured 4 weeks after insertion of the subcutaneous testosterone implant, between patients who reported ‘hair regrowth’ (329·79 ± 110·14 ng dL^−1^), patients who reported ‘no hair regrowth’ (300·16 ± 148·43 ng dL^−1^) and patients who had previously reported ‘no hair thinning prior to therapy’ (295·88 ± 104·72 ng dL^−1^) (Kruskal–Wallis χ^2^ = 1·25, d.f. = 2, *P* = 0·53) (Fig. 2).

Furthermore, no patient in this cohort of patients reported or listed ‘hair loss or thinning’ as a ‘negative or adverse side-effect from testosterone pellet therapy’ despite therapeutic serum testosterone levels four times greater, on average, than the upper limit of endogenous production (72 ng dL^−1^).

## Discussion

The results of our study raise important questions about the role of testosterone in causing hair loss in women. Several investigators have reported that women with hair loss are more likely to have elevated androgen levels or show an increased frequency of other features of androgen excess than women without hair loss. For example, Futterweit and colleagues^9^ reported that 38·5% of 109 women with hair loss had elevated androgen levels, and, in a series of 187 women, Vexiau *et al.*^10^ reported abnormal hormonal profiles, mostly of minor degree, in 67% of those with hair loss alone and in 84% of women who were also hirsute. In a series of 89 women presenting to a trichology clinic with hair loss, 67% showed ultrasound evidence of polycystic ovaries compared with 27% in a control group of 73 women, and 21% were significantly hirsute compared with 4% of controls.^11^ An increased frequency of insulin resistance has also been reported in women with hair loss.^12^ However, other investigators failed to find evidence of raised androgen levels in women with FPHL,^13^ and in all studies there is a variable proportion of women with hair loss who do not show clinical or biochemical signs of androgen excess. The results of clinical trials of antiandrogen therapy also provide no clear evidence for the role of androgens in FPHL. In a 1-year placebo-controlled study, finasteride 1 mg daily failed to prevent progression of hair loss in postmenopausal women with FPHL.^14^ Similarly, in a trial comparing cyproterone acetate with topical minoxidil, hair counts had fallen in the antiandrogen-treated group after 1 year, compared with an increase in hair counts in subjects using minoxidil.^15^ Subset analysis in this study did show a positive response to cyproterone acetate in women with irregular menses, suggesting an androgenic aetiology for hair loss in women with hyperandrogenism, but not in those without. Some support for this idea comes from an Italian trial which reported a small response to flutamide (but not to finasteride or cyproterone acetate) in hyperandrogenic women.^16^

In view of the widely held concept that androgens have a deleterious effect on scalp hair growth, the results of our study were unexpected. The study was not designed to investigate specifically the response of hair growth to androgen replacement therapy, and we recognize the limitations of a questionnaire in providing an objective measure of hair growth. To confirm our findings, formal assessment of hair growth, e.g. using methods such as a phototrichogram, would be needed. It is possible, for example, that a general positive effect on wellbeing of testosterone replacement misled subjects into believing that their hair growth had also improved. However, the fact that hair growth responded in the opposite direction to that expected and that patients reporting hair loss prior to therapy had lower testosterone levels than those not reporting hair loss does provide support for the veracity of the observation. Although a patient’s perception of hair thickness and texture is subjective, only one of 284 female patients was unsure of hair loss/thinning prior to testosterone therapy. Previous studies have shown that self-perception correlates with investigator assessment and photography (Dr T.L. Dawson, personal communication). In our experience, women are acutely aware of changes in their hair.

Significantly, no patient in this cohort, treated with continuous testosterone for over 1 year, reported ‘hair loss or thinning’ despite average serum testosterone levels of over 300 ng dL^−1^, four times the upper limit of normal for endogenous production, and sufficient to cause an increase in facial hair growth in the majority.

A possible explanation of our results could be that testosterone has anabolic as well as virilizing properties. Although these properties may overlap, the distinction is evident in men with a genetic deficiency of 5α-reductase type 2. These men have low circulating levels of DHT, but normal male levels of testosterone. They show incomplete genital development, reduced secondary sexual hair growth and absence of male balding, yet have a normal male pattern of muscular and skeletal development.^5^ Insulin, androgens and BMI (obesity) are related in women both with and without polycystic ovary syndrome.^17^ Insulin resistance, obesity and metabolic syndrome are associated with increased 5α-reductase activity and hair loss in both men and women.^12,^^18,^^19^ Insulin also stimulates testosterone biosynthesis, which may account for the higher testosterone levels seen in this group of patients.^20^ Perhaps insulin resistance and subsequent increased 5α-reductase activity (i.e. elevated DHT) impact hair loss and virilization, rather than testosterone. This is consistent with our findings – women who reported ‘no hair regrowth’ on testosterone therapy had significantly higher BMIs compared with patients who reported ‘hair regrowth’ on testosterone therapy.

We suggest, therefore, that our results indicate that testosterone has a positive anabolic effect on hair growth, which is distinct from a possible DHT-dependent deleterious role. The hair follicle is certainly a site of vigorous protein synthesis – the average human scalp produces around 13 km of hair per year. Androgen production in women declines with age and does not vary with menopause.^21^ A woman aged 40 years has half the mean plasma total testosterone of a 21-year-old.^22^ Mean hair density and hair shaft diameter show a similar age-related distribution in the female population^1^ and the fall in scalp hair density and hair shaft diameter, starting in the early to mid-thirties, parallels androgen levels much more closely than the common explanation relating female hair loss to postmenopausal oestrogen deficiency.

In conclusion, our results suggest that the effect of androgens on scalp hair growth in women is complex. Loss of hair may occur in hyperandrogenic states, possibly due to similar genetically determined DHT-driven mechanisms as occur in male balding, although the only evidence of this in our study was the lack of improvement in hair thinning in women with high BMIs. However, androgen deficiency may also lead to thinning hair due to loss of a more general testosterone-dependent anabolic effect on hair growth. This observation needs to be confirmed but potentially opens the way for a new approach to the treatment of female hair loss.

**What’s already known about this topic?**

- Androgens are implicated in causing scalp hair thinning in women.
- Circulating androgen levels in the female population decline from around 30 years of age.
- Scalp hair density and mean hair shaft diameter in the female population decline from early to mid-thirties.

**What does this study add?**

- Treatment with testosterone in women with symptoms of androgen deficiency improves scalp hair growth in a high proportion of those reporting hair thinning prior to treatment.
- Testosterone may have an anabolic effect on hair growth in women with symptoms of androgen deficiency.
- No subject reported scalp hair loss on testosterone treatment, casting doubt over the presumed role of testosterone in causing hair loss.
